# Contextualised Processing of Stimuli Modulates Auditory Mismatch Responses in the Rat

**DOI:** 10.1177/15500594241300726

**Published:** 2024-11-20

**Authors:** Jaishree Jalewa, Juanita Todd, Patricia T. Michie, Deborah M. Hodgson, Lauren Harms

**Affiliations:** 1 School of Psychological Sciences, College of Engineering, Science and Environment, 5982 University of Newcastle, Callaghan, New South Wales, Australia; 2 Hunter Medical Research Institute, Newcastle, New South Wales, Australia; 3 School of Biomedical Science and Pharmacy, College of Health, Medicine and Wellbeing, 5982 University of Newcastle, Callaghan, New South Wales, Australia

**Keywords:** mismatch negativity, animal model, context sensitivity, rats, order effects

## Abstract

Mismatch negativity (MMN), an auditory prediction error signal, is an enhanced response to unexpected (deviant) stimuli compared to expected (standard) stimuli. There is strong interest in MMN due to reliable findings of reduced MMN in schizophrenia. To interpret reduced MMN in schizophrenia, an enhanced understanding of the factors that influence MMN amplitude could lead to a better understanding of neural mechanisms underpinning the reduction. While several laboratories have observed mismatch responses (MMRs) in rodents, this study assesses how MMR is altered in more complex auditory sequences in rats. Prediction-errors are elicited in relation to “predictive” internal models of regularities. These internal models are updated dynamically when a regularity changes, but human MMN exhibits order effects when two regularities alternate; while deviants in both regularities elicit MMN (ie, the model updates) there is a slower build-up in MMN amplitude over time in the second encountered regularity type. We investigate whether order effects occur in rat MMRs. MMRs were studied to rare ascending and descending frequency deviations in awake, freely moving Wistar rats using wireless telemetry in both separate sequences (one regularity at a time) and in alternating sequences where regularities changed back and forth. The rat MMR did not show order effects, however, substantial MMRs occurred in response to both ascending and descending deviants in the alternating context but to the ascending deviant only when the same regularities were presented separately. The longer-term sequence structure altered prediction-error signalling in rat auditory system revealing a long term context sensitivity in internal models.

## Introduction

The Mismatch Negativity (MMN) component of the auditory event-related potential (ERP) is automatically triggered, without an active task or the requirement of attention, in response to unexpected deviant auditory stimuli among expected standard stimuli within an oddball sequence.^1,2^ MMN manifests as a negative deflection in the scalp-recorded voltage, typically occurring 100-250 ms after the deviance.^2–4^

MMN is an informative tool for understanding the brain's sensitivity to regularity and its contributions to perception and cognition.^5^ It provides a means to quantify the salience of unexpected stimuli, and is reliant upon intact dynamic pattern learning and formation of predictive internal models.^6–8^ Predictive processing is integral to goal-oriented behaviour, with the brain dynamically constructing predictions based on the content and quality of past information.^8^ When incoming sounds defy internal model predictions, MMN emerges as a neural signal of error and change detection, its magnitude modulated by “confidence” or precision in the predictive models.^4,9–12^ MMN has been suggested to be a precision-weighted error signal, such that predictions and prediction errors are modulated by the quality of evidence upon which they are based.^7,13,14^ For example, precision in the model will be higher for more stable environments, where confidence will accumulate each time predictions successfully match sound input. In addition, the precision weighted error signal will be large to deviants and even larger with increased rarity of deviants and increased departure of the stimulus properties from the model predictions.^7,13,14^

Internal models update rapidly when they repeatedly fail to account for sensory input. This has been studied by exploring how many times a deviation needs to repeat before a model adjusts to predict them which is inferred both from the reduction in response to this new repeating sound and the emergence of MMN to a future deviation from the new regularity.^15^ These dynamics have been studied in ‘roving paradigms’^16,17^ that enable systematic study of the deviant response amplitude following different numbers of standard repeats; each time a deviant occurs, it becomes the new repeating standard. Such paradigms confirm highly dynamic updating of the model predictions typically requiring only ∼3 repetitions of a new sound to form an updated prediction (more where the pattern is more complex^17^), with model precision increasing with consecutive repeats.^18^ However, studies in which a regular pattern alternates back and forth (ie, where two sounds alternate as standard and deviant at fixed intervals) exhibit order effects indicating that precision-weighting accumulates differently for the first regularity and the alternative.

In humans, order effects on precision-weighting were initially exposed by assessing MMN to the first and alternate deviant sounds in sequences in which the alternation rates were fast (0.8 min), medium (1.2 min) or slow (2.4 min^19^). The design question was whether models would show precision-weighting over stability periods and not just continuous standard trains such as in roving paradigms. The results confirmed that MMN amplitude to deviants was larger in more stable slower changing sequences but only for the first deviant. Subsequent investigations into why indicate that the regular alternation is essential to the effects^20^ and that this is associated with precision (indexed by MMN amplitude) accumulating more slowly in the alternate versus first-encountered regularity^6,13,21^ and see also.^22^ So far, at least three laboratories have reported this phenomenon of a *primacy bias* or a *first-impression bias* in predictive coding in human participants,^9,19,22,23^ which suggests that the way a sound is processed is influenced by initial learning about the probability, transitions, and/or importance of a sound.^5,7,13,19,24–28^ As the name suggests, ‘primacy bias’ refers to the impact of how the sequence starts, namely, the tendency for MMN to reach maximum amplitude quickly for the first DEV sound and stay large throughout a block, while the second DEV MMN typically starts smaller and increases as the block continues, showing a ‘bias’ of favouring accumulating confidence for the first DEV. This suggests that MMN amplitude is modulated differently for two sounds dependent on how the sequence begins. More specifically, it refers to how the probability of a given sound at the onset of a sequence can impact how the brain responds to it when it is later encountered at a different probability.^5,7,9,13,19,25,26^ This lasting impact of first learning suggests that the MMN can be biased by prior experience,^19^ and the subsequent experience is weighted in early relevance-filtering processes.^29^

The “suppression” of MMN has been attributed to the way that a sudden surprise (ie, a sudden increase in the occurrence of DEV events when the probabilities alternate) reduces confidence or precision in the predictions about the environment.^30,31^ When an existing model is no longer effective in accounting for the environment, new learning is triggered, and precision in the new model must build up over time, presumably reflected in the MMN amplitude as a precision-weighted signal.^9,19^ Todd et al also suggest that this may account for why the effect can be eliminated when a participant is first told about the alternating structure of the sequences (ie, the confidence/precision reaches maximum early in the block for both DEV tones). Without the surprise, the relearning seems to occur more.^25^ These findings of a primacy bias in humans suggest that factors that impact MMN size are not fully understood, and some mechanisms driving relevance-filtering are likely influenced by ‘top-down’ expectations. There is not yet an abundance of published studies focused on how such a bias is impacted by clinical conditions. One exception is a 2022 investigation into autism spectrum disorder, where the autism group was found to have MMN that was unaffected by the primacy bias, indicating that this group may have faster model updating compared to the typically developing group.^32^

Regarding the question of whether rats exhibit similar order effects in their Mismatch Responses (MMRs), previous studies have demonstrated that rats exhibit attributes of human MMN such as adaptation independence,^33^ sensitivity to N-methyl-d-aspartate receptor (NMDAR) disruption,^34^ deviance difference, deviant probability and stimulus onset asynchrony.^35^ It has also been established that rat MMR shows dynamic updating in the form of precision-weighting in terms of the deviant response being sensitive to the number of preceding standards.^36^ Understanding how initial learning affects precision-weighing in different contexts in rats will further validate the rat model as an analogue for human MMN and expand knowledge on the timeframes over which model evidence is accumulated and weighted in the underlying perceptual inference processes. To advance this aim, rat MMR was recorded both in traditional oddball sequences where there was a single regular tone and a single deviation, separately for an ascending frequency and a descending frequency, and to the same ascending and descending compositions concatenated into a regular alternating sequence. The traditional oddball sequences provide an index of MMR to a given deviant and deviant-probability within a highly stable environment, and the alternating sequence provides an index of the effects of order on dynamic model updating and precision-weighting. We hypothesise that if rat MMRs exhibit the same sensitivity to initial learning as human MMN, the MMRs would show a slower amplitude increase over time within alternating blocks for the alternate relative to first encountered deviation. That is, rats would have an increased response to the deviant presented first, compared to that presented second. In addition, the MMR to the second deviant would begin low, but become larger in the second half of the block as confidence in the predicted sequence accumulates.

## Methods and Materials

### Animals

Male and female Wistar rats (age: 3-4 months) underwent surgeries and were housed in pairs in standard open-top cages, in the temperature and humidity controlled Behavioural Sciences animal facility at the University of Newcastle, under normal light 12hr day-night cycle, and had *ad libitum* access to food and water.

### Surgery to Implant EEG Electrodes

Surgery was performed to implant stainless steel screw electrodes, similar to previous studies.^33,35^ Rats were anesthetised with isoflurane and fixed upon a stereotaxic frame, using blunt ear bars. Carprofen and buprenorphine were administered as analgesics prior to the start of the surgery. Six 0.9 mm burr holes were drilled at locations corresponding to the four recording sites (left/right rostral and caudal sites), the ground electrode, and the reference electrode dorsal to the cerebellum. The complete assembly of the connector along with six screws and their respective wires was fixed to the head using self-adhesive cement (SmartCem2, Dentsply, Australia) followed by temporary crown and bridge material dental cement (Integrity, Dentsply, Australia). The skin was then glued back onto the dental cement with Vetbond, followed by subcutaneous administration of sterile 0.9% saline to balance for any fluid loss during the surgery. Testing was performed after a week of post-surgery recovery.

### Sound Generation and Mismatch Response Testing

The auditory stimuli (frequency-modulated broadband tones, oscillating around a central frequency ± 3%) were generated using a custom program written in Presentation software (Neurobehavioral Systems, Inc.), and delivered through a speaker mounted on the roof of the experimental chamber.^33^ A sound meter (Bruel & Kjaer Model 2260) was used to calibrate the intensity of the free-field sounds to approximately 70 dB sounds in the range of 6636 Hz and 12 233 Hz. The broadband frequency-modulated tones were of 100 ms in duration, each centred on a particular frequency (eg 12 233 Hz) ± 3%, with 10 ms rise/fall times. It is noteworthy that previous studies in rats have shown an asymmetry in N54 responses being larger when the deviant frequency was higher than the prevailing standard repetition than when the same sounds were heard in reverse.^33^ Nonetheless, given the intention to assess change in N54 response as a function of deviant order, the centre frequencies were selected based on previous research showing that both produced robust evoked responses in a rat model^33^ albeit of different amplitudes.

MMRs were investigated in awake, freely moving rats. Using a reusable adhesive, a B-100mAh battery was connected to a wireless-10-channel telemetric headstage (Multi Channel Systems, Reutlingen, Germany). The wireless headstage was then inserted into the socket on the rat's head and the rat was placed in a 32 cm diameter circular plastic bucket (containing bedding) below the speaker through which acoustic stimuli were presented, in a sound-attenuating chamber (Med Associates, St. Albans, USA). Multi Channel Systems MCRack software was used to record the EEG data. Digitization of each channel of EEG data was performed at 2000 Hz (high pass filter 0.1 Hz, low pass filter 5000 Hz, voltage range ± 12.4 mV).^33^

### Experiment Design and Stimuli

**Figure 1.**
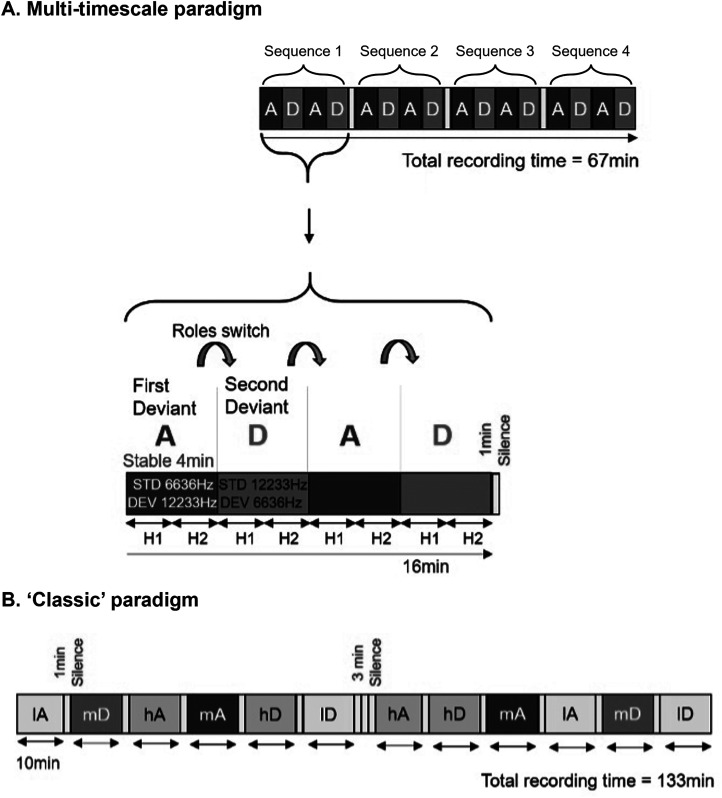
Illustration of alternating and traditional paradigms used to assess rat mismatch responses (MMRs). (A) A diagrammatic representation of the structure of alternating paradigm sound sequence developed for the current study. MMRs were examined in response to frequency deviants where the role of the deviant and the standard alternate. Here, the ‘High frequency deviant first’ sequence is depicted, in which block A (standard: 6636 Hz, 87.5% and deviant: 12 233 Hz, 12.5%) is presented at the onset, followed by block D after 4 min, when the probabilities of the two sounds are switched (*12 233* Hz at 87.5%, and 6636 Hz at 12.5%). This pattern continues so that the blocks ADAD were presented 4 times with 1 min silence breaks after every 4 blocks. On the second day of testing, these rats heard the ‘Low frequency deviant first’ block first (therefore heard DADA, etc). H1 (first half) and H2 (second half) show that each block was divided into halves for investigating half-effects. Half of the rats tested in the current study were exposed to this ‘High frequency deviant first’ design, whereas the other half of rats were exposed to a ‘Low frequency deviant first’ design. (B) A diagrammatic representation of the deviant probability manipulation paradigm as described in,^35^ the traditional paradigm. For a comparative analysis including ‘paradigm’ as a within-subjects factor, we selected only mid probability condition (*mA and mD*, similar to A and D, respectively, which is comparable to orange and purple in alternating paradigm) males and females combined) that were exposed to alternating paradigm at least 48 after testing on the classic paradigm. *lA:* low probability ascending oddball, *mA:* mid probability ascending oddball, *hA:* high probability ascending oddball, *lD:* low probability descending oddball, *mD:* mid probability descending oddball, *hD:* high probability descending oddball.

Rats were exposed to two sequence combinations: an ascending frequency deviant and descending frequency deviant. In each combination the probability of deviant and standard sounds were *P* = .125 and *P* = .875, respectively, all sounds were presented at a regular 500 ms stimulus-onset asynchrony, and all deviants were separated by a minimum of 3 standards. The ascending and descending combinations were presented in both traditional oddball compositions and alternating sequence compositions presented diagrammatically in Figure 1. Traditional oddball and alternating sequence compositions were presented over three different days and constructed as depicted in Figure 1.

The traditional oddball sequences were comprised of 2400 sounds and were presented as part of a probability manipulation which formed part of another study The probability of the deviant in each sequence is specified in the Figure 1 caption, the data utilised for the present analysis came from the medium probability blocks (mD in Figure 1) where the probability was the same as that in the alternating sequence blocks. The order of the probability and ascending and descending traditional oddball sequences was counterbalanced across rats, separately for each sex and all data was collected on a single day.

The alternating paradigm comprised four presentations of a four-block sequences with 1 min silence breaks in between each sequence presentation (as per Figure 1). Blocks contained 480 sounds after which a new block would commence with the former deviant becoming a new repeating standard with a minimum of five repetitions of this new standard at the beginning of the block. Rats were tested on the alternating paradigm over two days with half the rats hearing the sequence with the low frequency tones (6636 Hz) as the first deviant on day one (descending first, D block in Figure 1), and the high frequency tones (12 233 Hz) as first deviant on day two (ascending first, A block in Figure 1), and the other half of the rats hearing sequences in the reverse order across the days (counterbalanced across rats, separately for males and females).

### Data Extraction

**Figure 2.**
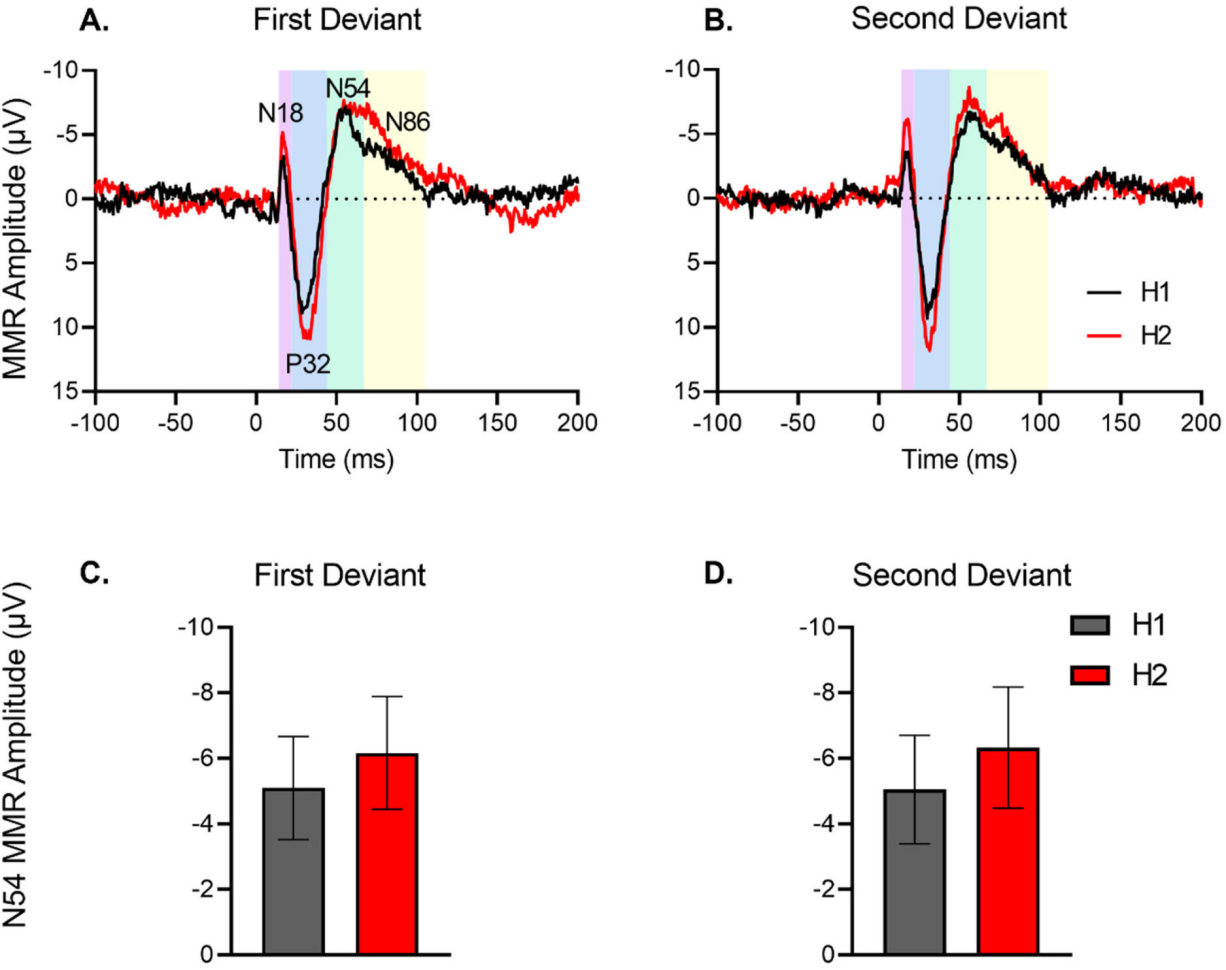
No primacy bias in rats (n = 16). Grand averaged difference waveforms for first deviant (A) and second deviant (B). The ERP plots show the MMR difference waveform (deviant-standard) for the first half (H1) and second half (H2) of blocks. Time window applied to extract the N54 mean amplitudes for the multi-timescale dataset (caudal: 43-70 ms; rostral 46-70 ms) is shaded as green. The represents the time windows for measuring mean amplitudes of N18, P32 and N86 (see Supplementary Information). (C-D) Mean amplitude (± standard error, SE) of N54 component of mismatch responses (MMR) for the first deviant (C) and second deviant (D) in first half (H1) and second half (H2) of blocks averaged for sequences 1-4. Absence of the primacy bias in rats was revealed by pairwise comparisons of halves for first deviant (H1 *vs* H2, *P* = .414; C) and second deviant (H1 *vs* H2, *P* = .260; D).

As described previously,^35^ data were processed off-line using EEGDisplay (version 6.4.1). After the pre-processing steps of bad channel rejection and artefact rejection prior to segmenting into epochs and averaging, the latency ranges of individual ERP components were determined based on the morphologies of the ERPs, taken from grand averages, and their mean amplitudes were extracted for each rat. The ERPs showed distinct components characterised by negative and positive peaks (N18, P32, N54, N86 – Figure 2A) but given previous findings of the N54 component of the rat MMR presenting as the most human MMN-like component,^35^ we focussed on whether there is a primacy bias effect on the N54 MMR measured as a mean amplitude between 43-70 ms (caudal electrodes) and 46-70 ms (rostral electrodes). Details of the analyses of other components can be found in the Supplementary Information. Once mean amplitudes were extracted separately for each recording location, using separate time windows for rostral versus caudal electrodes, data were then averaged across left and right electrode, rostral and caudal electrodes as done previously.^37^

### Statistical Analyses

All the analyses were performed using SPSS 28 and analysis of variance (ANOVA) with repeated measures was used to examine the mean amplitudes of the N54 component. Data was first inspected for inclusion by authors. One animal's data was excluded because of an equipment malfunction and another because of an earthing problem leaving usable data sets from 16 animals from the Alternating paradigm.

The principal aim of the study, to examine whether order effects are present in rodent MMN in alternating sequences, was serviced with N54 MMR as the dependent variable, and within-subjects factors of Frequency (High, Low), Deviant (First, Second), Half (H1, H2), and Sequence (first, second, third or fourth repetition of the sequences within a single session). Given that this is, to our knowledge, the first study using an alternating structure in rats, we also compared this data to that obtained in more conventional traditional oddball sequences that are highly stable and contain only one regularity and one deviant type. Rats for whom data was available on both paradigms (N = 13) were then compared in a repeated measures ANOVA with N54 MMR as the dependent variable, and within-subjects factors of Frequency (High, Low) and paradigm (Traditional, Alternating). For both paradigms, N54 was also measured in deviant and standard ERPs using the same latency window as for MMRs to enable clarifications of whether any observed differences were due to differences in standard or deviant responses or both. Key methodological differences between both the paradigms are explained in the Table 1.

**Table 1.** Summary of same/different features in the design of traditional oddball versus alternating paradigms.

Feature	Traditional Oddball paradigm	Alternating paradigm
Blocks in the sequence	Random	Alternating
Block length	Long (7-10 min)	Short (4 min)
Counterbalanced	Over 1 day	Over 2 consecutive days
Silent breaks between blocks	1 min	No silent breaks
Sounds	6636 Hz and 12 233 Hz	6636 Hz and 12 233 Hz
Probabilities	87.5% for standard 12.5% for deviant	87.5% for standard 12.5% for deviant
Stimulus -onset-asynchrony	500 ms	500 ms
Sound presentation	Overhead speaker, 70 dB	Overhead speaker, 70 dB
Trial count for deviant averages	300 per deviant	Each Sequence: 120 per deviant Block Half: (60 × 4) 240 per deviant Each Order: (120 × 4) 480 per deviant Overall: (480 × 2) 960 per deviant

## Results

### The Rat MMR in the Alternating Paradigm

Although half of the rats tested in the alternating paradigm study were exposed to the ‘High frequency deviant first’, whereas the other half of rats were exposed to a ‘Low frequency deviant first’ (see Figure 1 caption), after loss of data for technical reasons, 6 animals heard the High frequency Deviant first and 10 heard Low frequency Deviant first. The between-subjects order of exposure to the deviant was not included in the analysis. The repeated measures ANOVA on N54 amplitudes in the alternating paradigm indicated a significant main effect of sequence only (sequence effect: *F*_(3,45) _= 4.54, *P* = .013) with effects of frequency (*F*_(1,15) _= 0.105, *P* = .740), deviant (*F*_(1,15) _= 0.003, *P* = .955), and half (*F*_(1,15) _= 2.145, *P* = .164) being non-significant with no significant interactions. The ERP responses are presented in Figure 2 with MMR to the first deviant (a) and alternate or second deviant (b) plotted separately for each block half, but averaged over frequency and the associated mean amplitudes for these presented in (c) and (d), respectively. As evident in Figure 2, the size of the MMR did not increase significantly from the first to second half of blocks and did not exhibit differential change for the first and alternate deviant, suggesting the absence of a primacy bias in the rats (Deviant * Half: *F*_(1,15) _= 0.014, *P* = .909, Figure 2). Although N54 amplitudes were slightly smaller for the first half for both deviants (see Figure 2c and d) this change was not significant indicating very dynamic model updating and a rapid increment in precision-weighting in both blocks.

**Figure 3.**
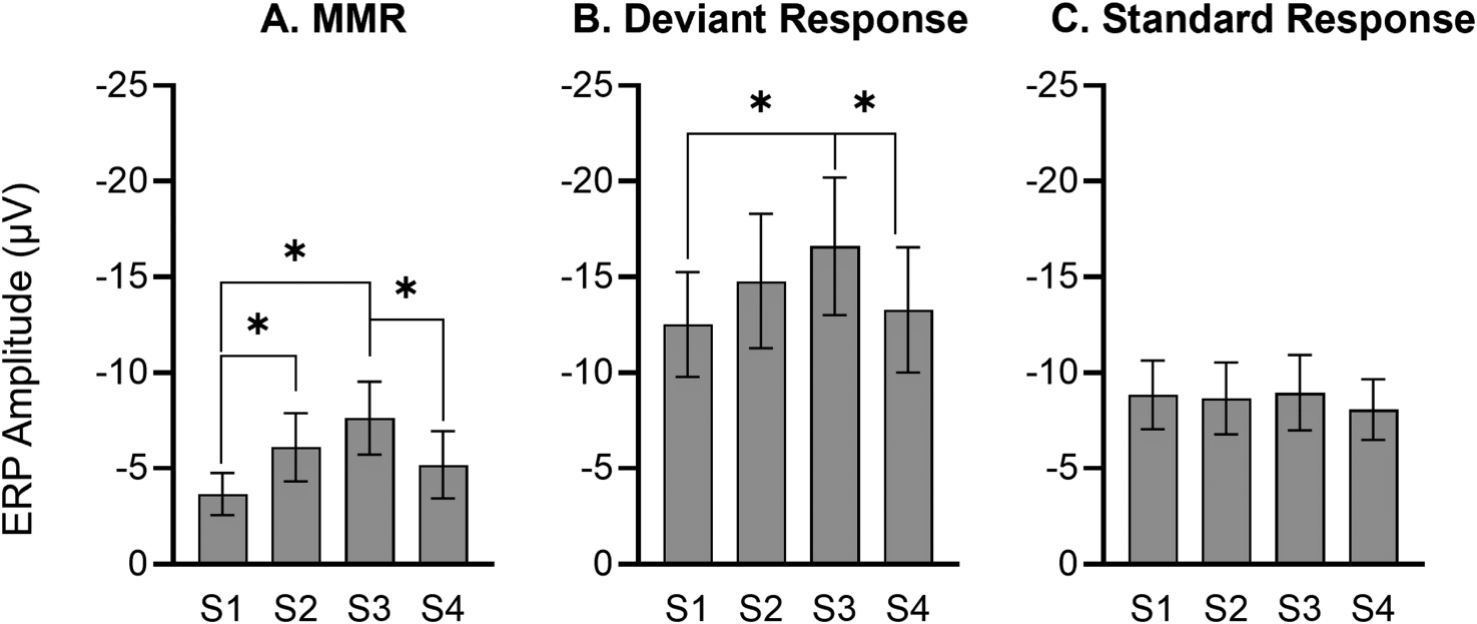
Sequence main effect (n = 16). (A) Mean amplitude (± standard error, SE) of N54 component of mismatch responses (MMR) for first, second, third and fourth repetition of the sequences. The MMR amplitude gets larger from first presentation (S1) to third presentation (S3) linearly, and then decreases back nearly to the size of MMR in the first presentation (S1). Mean ERP amplitude (+ standard error, SE) for first, second, third and fourth repetition of the sequences in response to deviant (B) and standard (C) stimuli. Response to deviant stimuli increases from S1 to S3 and then declines back in S4, but no change in response to standard stimuli over sequence presentations.

The main effect of sequence on N54 is evident graphically in Figure 3 where the average amplitudes are collapsed over frequency, deviant, and half. MMR amplitude in rats increased from first presentation (S1) to third presentation (S3), and then decreased back to the size of MMR in the first presentation in S4 (Sequence effect: *F*_(3,45) _= 4.154, *P* = .013). This result was revealed by pairwise comparisons of sequences (Figure 3A, S1 *vs* S2, *P* = .039; S1 *vs* S3, *P* = .004; S3 *vs* S4, *P* = .037). The increase in MMR amplitude from S1 to S3 was likely driven by increased responses to deviant stimuli when presented in S1 to S3 as deviant amplitudes showed trend-level effect of sequence (*F*_(3,39) _= 2.649, *P* = .062), revealed by pairwise LSD comparisons of sequences (Figure 3B, S1 *vs* S3, *P* = .027; S3 *vs* S4, *P* = .007). There was no significant effect of sequence on standard responses (Figure 3C*, F*_(3,39) _= 0.856, *P* = .472).

### The Rat MMR in Alternating Versus Traditional Oddball Paradigms

**Figure 4.**
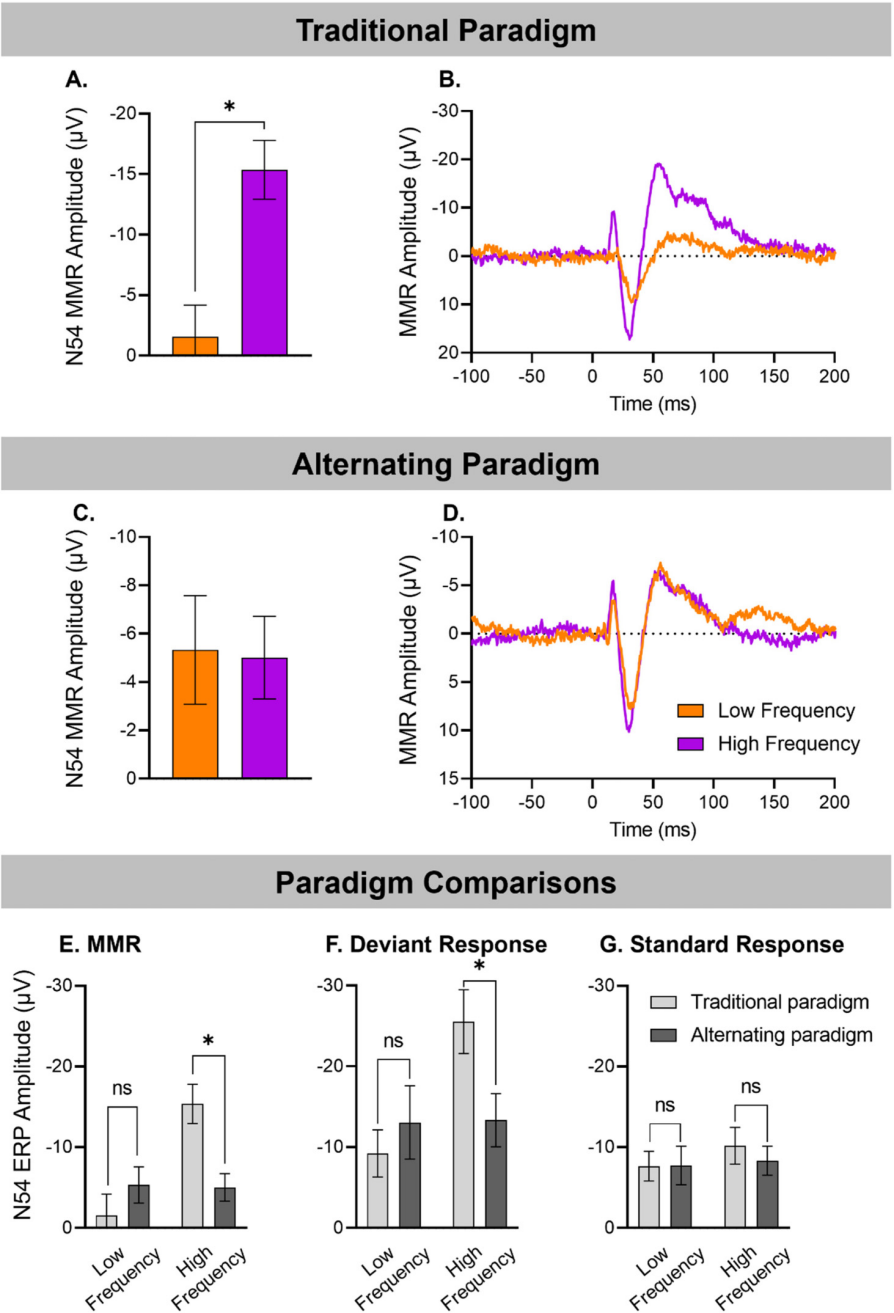
Frequency effects in rats tested on two separate paradigms (n = 13). MMRs are shown for traditional paradigm (A, B), alternating paradigm (C, D), and MMR, deviant and standard responses are shown comparing both paradigms (E-G). In the traditional paradigm, N54 MMR size was highly dependent upon stimulus frequency (*n *= 13, Frequency effect: F_(1,12) _= 12.25, *P* < .004, as seen in the (A) mean amplitude MMRs (±SE), and (B) grand averaged waveforms in response to low frequency and high frequency. In the alternating paradigm, stimulus frequency did not affect N54 MMR size (Frequency effect: F_(1,12) _= 0.032, *P* = .860), as seen in the (C) mean amplitude MMRs (±SE), and (D) grand averaged waveforms. When comparing the traditional versus alternating paradigms, (E) mean N54 MMR amplitude (±SE) did not differ between the alternating and traditional paradigms for low frequency stimuli (*P* = .308), but smaller in the alternating paradigm for high frequency stimuli (*P* = .005). (F) Low frequency mean amplitude responses (±SE), to deviants did not differ in the traditional and alternating paradigms (*P* = .974) but for high frequency, the deviant amplitude was reduced in the alternating paradigm (*P* = .015), (G) but no difference between standard responses in the two paradigms for either low or high frequencies.

Data from 13 animals was available for both the Alternating and Traditional Oddball paradigms. The MMR response to ascending and descending deviants in the traditional oddball paradigm and that averaged over all occurrences of each deviant in the alternating paradigm are presented in Figure 4B and D, respectively, with associated mean amplitudes in 4A and 4C, respectively. As reported above, there was no significant effect of Frequency on N54 MMR amplitude in the alternating paradigm (F_(1,12) _= 0.32, *P* = .860) with clear responses to both the low frequency or descending deviant (−5.33 ± 2.24 μV), and high frequency or ascending deviant (−5.01 ± 1.71 μV) and both exhibiting the typical morphology observed previously in recordings from our lab.

When N54 was compared across the two paradigms there was a significant Frequency by Paradigm interaction effect: (*F*_(1,12) _= 8.40, *P* = .013). As evident in Figure 4, this interaction is due to a very clear difference in N54 MMR amplitude for the ascending/high frequency deviant and descending/low frequency deviant in the traditional oddball paradigm (*P* = .004), but no difference in N54 response amplitudes for the two deviants in the alternating paradigm (*P* = .860). Furthermore, the low frequency N54 did not differ significantly in the alternating compared to the traditional oddball paradigm (*P* = .308), while the high frequency N54 was significantly larger in the traditional oddball paradigm (*P* = .005).

Standard and Deviant responses in alternating and traditional paradigms.

The paradigm-induced changes in the size of N54 MMR were associated with significantly reduced responses to high frequency deviants in the alternating paradigm, relative to the traditional oddball paradigm (Stimulus * Frequency * Paradigm interaction (*F*_(1,23) _= 8.398, *P* = .013). Pairwise comparisons revealed only a significantly reduced amplitude to the high deviant response in the alternating paradigm relative to the traditional oddball paradigm (*P* = .015 Figure 4G). The increase in the low frequency deviant N54 amplitude (Figure 4F) was not significant (*P* = .451). It is apparent from Figures 4F and 4G that the alternating paradigm resulted in very similar deviant and standard amplitudes for both low and high frequency stimuli in contrast to the traditional paradigm, resulting in similar MMR amplitudes for both high and low frequencies. Additional multiple comparisons of standards and deviants from the two paradigms (Table 2) revealed that the deviant response was significantly larger than the standard response for both low (*P* = .035) and high (*P* = .013) frequencies in the alternating paradigm whereas this comparison was significant only for high frequency stimuli (*P* < .001) and not for low frequency (*P* = .558) in the traditional paradigm.

**Table 2.** The Mean Amplitudes and Standard Errors (in Parentheses) for the N54 Period for Each Sound as a Repetitive Standard and Rare Deviant in the two Sound Sequences Paradigms (*n *= 13).

	Traditional	Alternating
Low Standard	−7.66 µV (±1.83)	−7.74 µV (±2.39)
Low Deviant	−9.23 µV (±2.92)	−13.064 µV (±4.53)
High Standard	−10.18 µV (±2.73)	−8.33 µV (±1.79)
High Deviant	−25.54 µV (±3.94)	−13.34 µV (±3.29)

## Discussion

In human MMN studies, an order effect, where the MMN amplitude is influenced by the order of sounds in a sequence, has been observed.^7,24^ However, we did not find a significant order effect in rats, suggesting a lack of sensitivity to this order-dependent bias. Further, unlike findings in humans where MMN amplitude constantly declines across multiple presentations of oddball sequences,^13,24,38^ our findings in rats show an increase in the MMR across presentations. Both observations therefore suggest some differences in the pattern of precision-weighting over time in rat MMR versus human MMN. We also replicated previous observations of differential sensitivity to ascending versus descending frequency deviants in separate traditional oddball sequences where N54 amplitudes were much larger for ascending arrangements,^33^ but not when these very same sound compositions were heard within a concatenated alternating structure. The effect of paradigm on frequency asymmetry in the rat MMR seems to have been mainly due to effects on the ascending deviants where the advantage of prolonged stability in the traditional sequence was evident in the N54 amplitude. This was not the case for the descending deviant responses, which did not differ significantly in the two sequence types. There was some evidence that N54 to the descending deviant was enhanced in the alternating paradigm but this was not significant. Nonetheless, the slightly larger N54 to the descending deviant no doubt contributed to the loss of MMR frequency dependency in the alternating paradigm.

The lack of an order effect where the MMR to the first deviant was larger than that for the second deviant, and not observing the second deviant growing over time shows that at least in this respect, the rat MMR does not mirror the features of human MMN. Our work has previously shown that that rat MMR mirrors several other aspects such as adaptation independence,^33^ sensitivity to N-methyl-d-aspartate receptor (NMDAR) disruption,^34^ and other sequence characteristics.^35^ The unexpected finding of a lack of order effects in the current study may indicate that we have found the limit for which rat MMRs match human MMN or that different stimulus conditions are needed for the effect to be observed. The MMRs observed in the current study were slightly lower in those observed using similar methodologies previously,^35,37^ but such effects may be due to the fast alternating of the sequences in the current study. At the beginning of the experiment, all animals were exposed to a high-deviant first experiment on one day, and the low-deviant first experiment the other day, and the order in which they were exposed to these was evenly distributed across animals. However, some subjects were not included in the analysis due to poor recording quality, which left the sample of animals skewing towards those exposed to the Low Deviant First on day one of testing (n = 10), compared to a smaller number exposed to the High Deviant First (n = 6). Because of this, we couldn’t examine any kind of overarching primacy effects where the first stimuli presented as a deviant would influence MMRs up to several days in the future.

The alternating sequences have exposed some differences between the rat MMR and human MMN in terms of the apparent speed of model updating and precision-weighting in the new models when a regularity suddenly changes. In humans, exposure to four-block sequences with this alternating structure reveals differences in the way that the deviant response is weighted over time within the blocks. For sounds encountered as the first deviant, the response is equally large over early and later portions of the block while the deviant response and MMN are significantly smaller during the early first half of a block than later in the block for the alternative deviant. This has been shown for sounds of different duration,^26^ spatial location, and frequency.^6,29^ In humans, most studies have been conducted using sounds of two different durations where it was shown that these different patterns of response weighting persist when sequences are repeated,^13,24,38^ as in this study, but they disappear in older aged adults,^39^ and they also disappear if the participant's attention is absorbed in a cognitively demanding concurrent task.^25^ The absence of order effects on MMR in rats in the present study is therefore not entirely inconsistent with human MMN, but where order effects are absent in humans, the results show a significantly smaller MMR during the early first half of a block, compared to later in the block, for both the first and alternate deviant (a main effect of Half). While there is a visible trend for N54 MMR to increase over half in the present study, this was not significant, leaving the interpretation open to the potential for faster model updating and accumulation of precision in rats as opposed to humans.

What is quite different in the rat N54 MMR is the tendency for the amplitude to increase with repeated exposure to the sequences, at least from the first to the third sequence. This contrasts with sequence repeat effects in humans which have consistently shown an overall decrease in MMN size with repeating sequences.^13^ There are two possible explanations for the pattern observed in rat MM.The first is that repeated exposure to the sequences enables the rats to fine-tune the precision of the model and the error representations. Frequency receptive fields do change with exposure to sound, and furthermore they change in a manner that can interact with the relevance of sound.^40^ This links the first explanation with a possible second explanation: the potential enhancement of the N54 MMR over time if the rats are attending to the sounds. The potential augmentation of the N54 MMR by attention could also perhaps then explain the decline in the MMR by the fourth sequence if this attention has waned. In summary, it is not possible to know precisely why the N54 MMR changes over sequence repeats, but it is nonetheless a clear difference in how the rat and human deviance responses are changing over time for sequences of this kind.

Previous rat studies using a ‘traditional’ paradigm showed the typical frequency-dependent asymmetry with larger MMRs in response to ascending frequency deviant stimuli, with descending frequency MMRs very small or sometimes completely absent.^33,35,41^ It has been suggested that the tendency for rat MMRs to be larger in response to frequency increments (compared to decrements) is due to the relative rarity of high-frequency sounds in the natural environment, thus making them highly salient to the animal.^42^ Similarly, we have previously posited that the high salience of high-frequency sounds could be related to the survival-value of these stimuli, being closer in frequency to the ultrasonic vocalisations rats use to communicate alarm signals to each other.^33^ Contrary to these previous findings however, our study using the alternating paradigm revealed similar MMR sizes for both ascending and descending deviants, challenging the notion that ascending deviants are generally more salient than descending ones.^42^ A possible explanation for the absence of this asymmetry is that when the sounds are actually informative about sequence structure, the system becomes equally sensitive to both deviations.^5^ This interpretation would suppose that the rats were sensitive to this emergent longer-term regular structure (ie, regularities change over time) which requires further consideration. Others have suggested that the frequency asymmetry in rat MMR represents a naturally higher salience for an ascending deviant, and that this larger MMR can be altered through exposure to sound that contradicts this empirically rare differential.^42^

In a study from Shiramatsu et al^,^^42^ the ascending-larger-than-descending asymmetry in MMR seen in naïve rats was eliminated in rats who were first exposed to sequences in which the higher frequency sound rarity was systematically decreased. Appetitive and aversive conditioning conditions paired with auditory stimuli, tended to augment the MM. In the present study, both the traditional oddball and the alternating testing paradigms involved equal rarity for ascending and descending frequencies over the testing session; a scenario that preserves the empirical asymmetry. It is therefore proposed that the elimination of frequency asymmetry in the present study may be attributable to the importance of the changes in sound tendency to predicting the longer-term sound structure, or alternatively the tendency for attention to be drawn to these sounds at the critical change-over points in regularity where the former deviant sound begins to suddenly repeat.

The findings from this study show that the paradigm chosen is not sufficient to produce order effects in the rodent MMR that mirror those in humans. However, given our previous reports that rats exposed to risk factors for schizophrenia (maternal immune activation and adolescent cannabinoid exposure) exhibit MMR deficits in a highly context-dependent manner,^37^ it is possible that the paradigm used here may be applied to schizophrenia models such as developmental or genetic risk factor models or pharmacological models using NMDA antagonists. In addition, this study has shown how the sequences can be altered to overcome frequency asymmetry. While the opportunity to unlearn natural salience may be one way to overcome frequency asymmtetry,^42^ having sound tendencies be the means of extracting a discoverable longer structure may be another. Indeed, four-day old neonates also show a sensitivity to sounds arranged in alternating sequences that is not seen in traditional oddball versions of the same compositions.^43^ Future studies are required to replicate the present findings within a fully counterbalanced design and to more systematically test the potential importance of longer-term structures to the sensitivity to locally deviant sounds.

Our findings provide new data on the differences between rat MMRs and human MMN in response to sound sequence deviations. While humans show clear order effects and a decline in MMN amplitude over time, rats exhibit no significant order effects and, intriguingly, an increase in MMR amplitude across sequence presentations. This suggests species differences in the precision-weighting of sound sequences and potentially faster model updating in rats. Additionally, the elimination of frequency asymmetry in the alternating paradigm challenges previously held assumptions about the salience of ascending deviants, indicating that context and regularity play a significant role in shaping auditory deviance responses. These findings have implications for future studies on schizophrenia models, particularly in understanding how sequence structures could modulate MMR deficits. Further studies are necessary to explore the full implications of these differences and to determine the potential applications of these paradigms in other contexts, such as developmental studies or clinical conditions.

## Supplemental Material

sj-docx-1-eeg-10.1177_15500594241300726 - Supplemental material for Contextualised Processing of Stimuli Modulates Auditory Mismatch Responses in the Rat Supplemental material, sj-docx-1-eeg-10.1177_15500594241300726 for Contextualised Processing of Stimuli Modulates Auditory Mismatch Responses in the Rat by Jaishree Jalewa, Juanita Todd, Patricia T. Michie, Deborah M. Hodgson and Lauren Harms in Clinical EEG and Neuroscience
