# Supplementary material for: Contextualised Processing of Stimuli Modulates Auditory Mismatch Responses in the Rat
Source: Clin EEG Neurosci. 2024 Nov 20;56(1):35–45. doi: 10.1177/15500594241300726 (PMC11664890; doi:10.1177/15500594241300726)
Supplement: sj-docx-1-eeg-10.1177_15500594241300726 - Supplemental material for Contextualised Processing of Stimuli Modulates Auditory Mismatch Responses in the Rat [file sj-docx-1-eeg-10.1177_15500594241300726.docx]

**Supplementary information.**

**Analysis of N18, P32 and N86 in the Alternating paradigm**.

N18, P32 and N86 were measured as mean amplitudes over 31 – 21.5 ms, 22.5 - 42 ms and 67 - 105 ms respectively.

Each average amplitude was analysed in separate repeated measures ANOVAs with factors of Deviant (first vs second), Frequency (Low vs High), Half (First half vs second Half), and Sequence (S1, S2, S3, S4).

For N18, Half was significant (F(1,15) = 9.68, p=.007. N18 MMR amplitude was larger in second half (-3.547µV) than the first half (-1.783 µV) evident in Figure 2 A and B. In addition, both the Sequence main effect and a Half x Sequence interaction were significant (F(3,45) = 3.06, p = .049) and (F(3, 45) = 3.454, p = .034) respectively. Pairwise comparisons revealed that MMRs in the first half of blocks peaked at Sequence 3 (Sequence 3 > Sequence 2, p = 0.027). For the second half of blocks, N18 MMRs peaked at Sequence 2 (Sequence 2 > Sequence 1 p < 0.001; Sequence 2 > Sequence 3 p = 0.014, Figure 1S).

For P32, Half was again significant (F(1,15) = 5.04, p = .040. P32 was larger in second half (6.93 µV) than the first half (5.30 µV), again evident in Figure 2. There was also a Sequence main effect (F(3,45) = 3.96, p = .019): P32 increased from Sequence 1 to 2 (p = 037) with a further increase for Sequence 4 (p = .010) relative to 1 (Figure 2S).

Analysis of N86 revealed a Deviant x Sequence interaction only (F3,45) = 3.041, p = .044). For the First Deviant, N86 MMRs peaked at Sequence 2 (Sequence 2 > Sequence 4 p = 0.042). For the second deviant, N86 MMRs peaked at Sequence 4 (Sequence 4 > Sequence 1 p = 0.018, (Figure 3S).

Although each component was sensitive to repetition of the sequence either as a main effect or interaction in the Alternating paradigm, like N54, neither of the earlier components (N18, P35) nor the later component (N86) in rats showed sensitivity to an order-dependent bias.


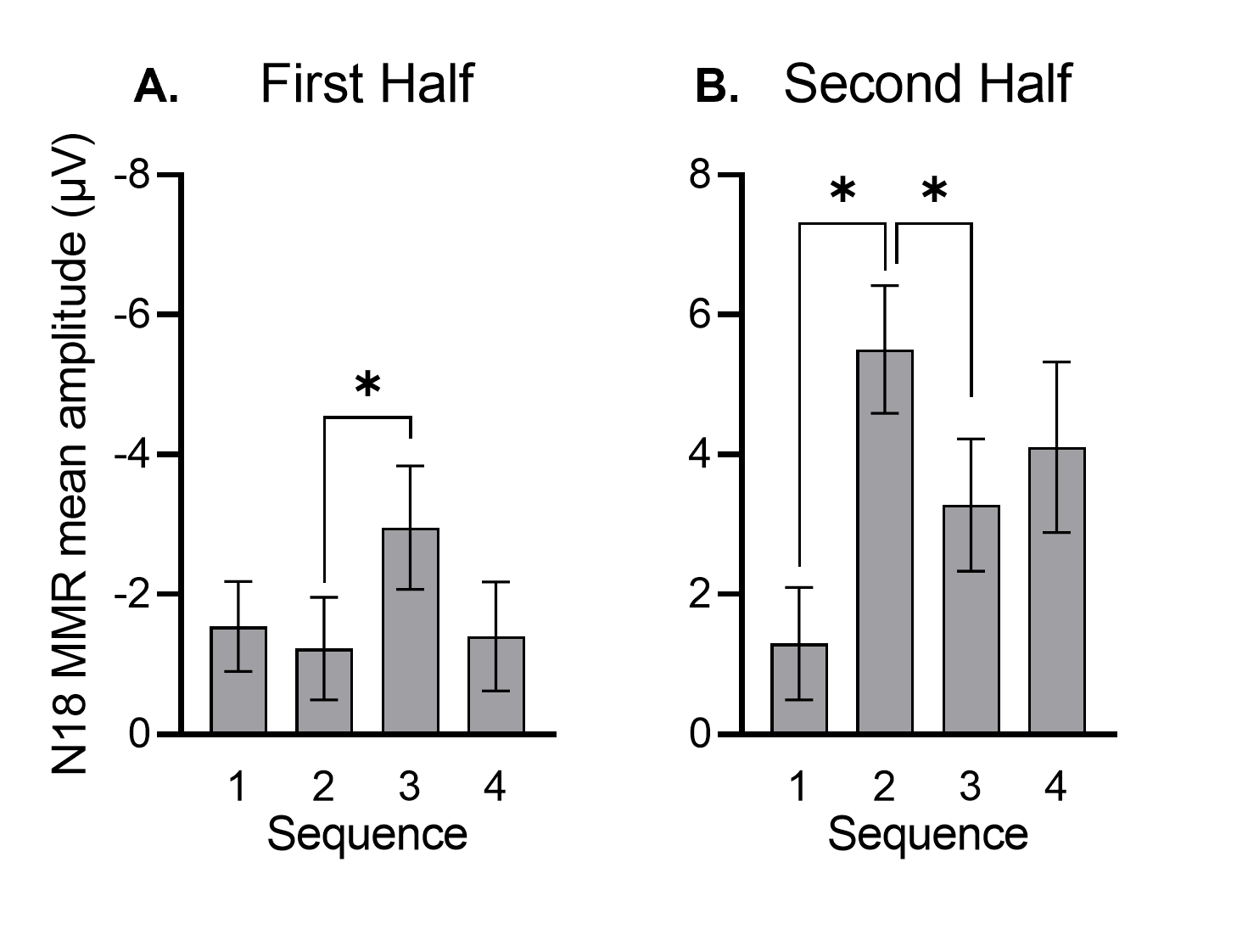


**Figure 1S. Sequence by Half interaction effect on N18**. Mean amplitude of N18 + Standard Error (S.E.) for the first, second, third and fourth repetition of the sequences in the first half (A) and second half (B).


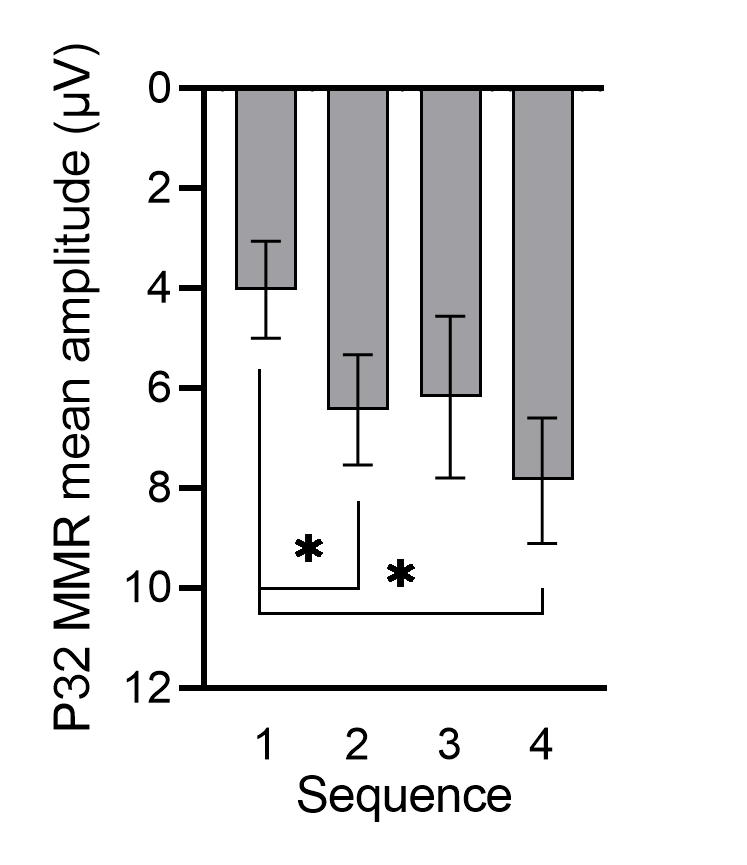


**Figure 2S. Sequence main effect on P32**. Mean amplitude of P32 + Standard Error (SE) for the first, second, third and fourth repetition of the sequence.


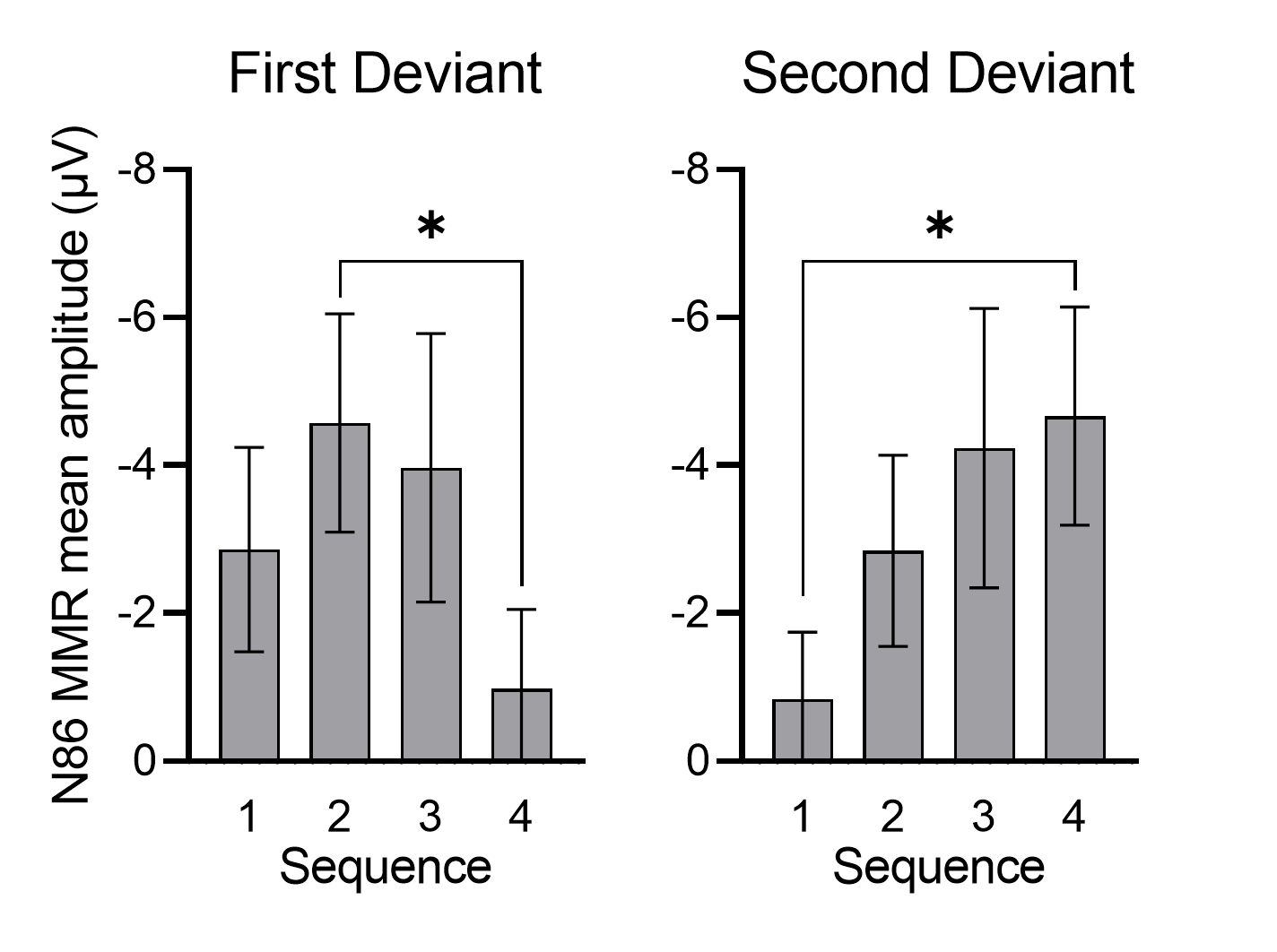


**Figure 3S. Deviant x Sequence effect on N86**. Mean amplitude of N86 + Standard Error (SE) for the first, second, third and fourth repetition of the sequences for the first deviant on the left hand side and second Deviant on the right hand side.
